# Paraphilia without symptoms of primary psychiatric disorder: a case report

**DOI:** 10.1186/s13256-023-03774-8

**Published:** 2023-02-13

**Authors:** Nicholas Tze Ping Pang, Ruziana Masiran, Aishah Siddiqah Alimuddin

**Affiliations:** 1 Faculty of Medicine and Health Sciences, University Malaysia Sabah, Jalan UMS, 88400 Kota Kinabalu, Sabah Malaysia; 2 Faculty of Medicine and Health Sciences, University Putra Malaysia, 43400 Serdang, Selangor Malaysia

**Keywords:** Paraphilia, Exhibitionism, Voyeurism, Frotteurism, compulsive sexual behavior

## Abstract

**Background:**

Paraphilias are recurrent and arousing fantasies, thoughts, and behaviors that cause distress to sufferers and surrounding people. This case report details the challenge of managing multiple paraphilias with compulsive sexual behavior.

**Case presentation:**

A 48-year-old Malay man presented with compulsive sexual behavior, encompassing voyeuristic, frotteurism, and exhibitionistic behavior, increasing progressively over the years, with accompanying overvalued ideas of erotomania. Despite the high level of dysfunction occupationally and socially, there were no apparent psychotic, manic, or depressive symptoms. An organic workup was unremarkable, and he was diagnosed with multiple paraphilias. Treatment with selective serotonin reuptake inhibitors was commenced, and psychologically he was managed with techniques specific to compulsive sexual behavior.

**Conclusion:**

Though rare in the literature, both paraphilic disorders and compulsive sexual behaviors are very distressing to sufferers and their families alike, and thorough biopsychological investigations are essential to ensure reversible causes are not overlooked.

## Introduction

Paraphilic disorders refer to recurrent, sexually arousing fantasies, urges, or behaviors involving inanimate objects, children, or nonconsenting adults. They can become physically and psychologically debilitating, harming affected individuals and their partners. They can also contribute to reputational risks such as humiliation to oneself or affected partners. There is thus a prerogative to identify them and associated underlying organic or psychological etiologies ensuring treatment for reversible risk factors. This case describes multiple paraphilias in a person without other discernible psychiatric disorders, which can be challenging to identify and manage in a culturally reserved Asian society.

## Case presentation

Mr. A is a 48-year-old Malay male working as a dispatch rider. His family brought him in after he molested a worker in his sister’s cafe. He had walked into the kitchen and brushed against her buttocks without provocation, followed by having dry sex with her without taking off his clothes. It was noted that this was not his first time doing so, as his sister had received several complaints about his touching staff members while working on different occasions.

This was on a background of an increase in sexually disinhibited behavior since 1999. He had married his wife in 1996, but 3 years into the marriage, he had started to be more sexually demanding toward his wife. He repeatedly asked for sex, sometimes demanding it for an entire week, causing the wife to require a few stitches in the vaginal area on one occasion. He then became dissatisfied with his wife as she stopped obliging to his sexual requests and thus started to ask other women he knew to satisfy his sexual needs. This led to his marriage being dissolved in 2009.

Following his divorce, he started to show exhibitionistic tendencies and flashed to a staff nurse, resulting in a police report being lodged. However, his family bailed him out. There were also increasing tendencies of touching others where he had allegedly fondled others at the workplace and in public spaces. He was fired from one job as a staff member had alleged he had touched her breasts.

He also had increasing stalking behaviors whereby he would ogle at passing women near his sister’s cafe in a pronounced fashion and then strike up conversations with them and give them his number after what he described as a short period of getting to know them better to ascertain their compatibility with him. He sometimes follows women passing by in the neighborhood until they notice him stalking them. The women would scream, and he usually took flight before anyone could apprehend him; thus, no police cases had ever resulted from this. He, however, did not acknowledge any of the above incidents and regarded them as accidents.

In his personal space, he had increased pornography use. He started to amass a collection of pornographic DVDs at home and admitted to watching them at least twice a week at home. He also owns blow-up dolls and would masturbate into them twice weekly whenever the desire overtook him. When this was insufficient, he would go to local prostitutes and even on a trip to neighboring countries to engage in various acts such as lap dancing, touching call girls, and having sexual intercourse with them.

He also speaks of possible marriage to certain celebrities he thinks are compatible with his lifestyle ideas. He had sent gifts, for example, rings, perfume, and jewelry, to a famous local celebrity 10 years ago to attract her attention to become his partner. This escalated into him texting her, standing outside her house, and waiting for her to come out so she could talk to him. He even went to her house to see her on the pretext of delivering gifts as a despatch rider. In 2011, he waited outside her house for so long that she finally became concerned and rang the police. He acknowledged that the celebrity would not reciprocate and never believed she was in love with him. He described himself merely as a loyal fan who harbored wishes of marrying her. His ideas were shakeable and not firmly held or fixed. He justified what he did to her as something a male would do to fulfill his interest. He later shifted his attention to another celebrity. He had gone to her boutique and told her father that he wanted to get married to her. This again resulted in a police report being made, but no further action was taken as he had left the vicinity.

His sexually driven activities used up a large proportion of his income, and he frequently borrowed money from his family for food and daily living. He was engrossed in pursuing sexual activities to the point of excluding other activities, and there was evident social and occupational dysfunction. He denied any elements of sadism, masochism, transvestic fetishism, or other fetishes. All his sexual acts were performed under full knowledge of the consequences of his actions and their meaning and implications.

Throughout all this, there were no apparent depressive or manic symptoms elicited. He did not display any perceptual disturbances, and none of his ideas was ever held to delusional intensity. According to collateral history from family members, no disorganized speech or behavior was noted at any point. He did not feel that any of his actions were under the control of an external agency. There was no feeling that his thoughts were inserted, withdrawn, or broadcasted. At no point did his ideas present themselves as intrusive obsessional thoughts, and he did not feel any need to relieve anxiety via a compulsion.

He had no medical or surgical illnesses or substance use on further history. There was no family history of any psychiatric illness. He got divorced in 2009 after 13 years of marriage, with his symptoms noticeable only after the third year of his marriage. Two identifiable major precipitating events for his symptoms include the loss of his mother a year before the symptoms started and the loss of his job in the same year, resulting in him feeling sad and, simultaneously, ashamed as his wife had to become the family’s breadwinner. This allows a simple formulation that he had lost control over his life in many ways, especially financially (the traditional preserve of a conventional male). Thus, the increased sexual desire was an attempt to reassert his masculinity and dominance physically. Premorbidly he was a quiet man who usually kept to himself and did not have many friends.

Physical examination, including neurological examination, was unremarkable. The mental state examination was equally unremarkable. Various investigations (Table 1) failed to reveal any organic cause.

**Table 1:** Investigations done and other ideal suggested investigations that could be done

Investigations done	Other suggested investigations that could be done
1) Routine blood—full blood count, renal profile, thyroid function test, and liver function tests: normal 2) Infective screening—VDRL, hepatitis B/C, and HIV: negative 3) Prolactin level: 866 ng/mL (upper normal) 4) Testosterone level: normal 5) Contrast-enhanced CT brain: no significant findings 6) Mini-Mental State Examination (MMSE) score: 29/30 7) Frontal assessment battery: no obvious deficits 8) Sexual Addiction Screening Test score: 11 (close to a cutoff point indicating sexual addiction)	1) Phallometry (penile plethysmography) 2) Multidimensional Inventory of Development, Sex, and Aggression (MIDSA) 3) Multiphasic Sex Inventory (MSI)

He was diagnosed with a paraphilic disorder of three types (exhibitionism, voyeurism, and frotteurism), following a consensus obtained from several psychiatrists (Table 2). On those grounds, a selective serotonin reuptake inhibitor (SSRI), escitalopram, was initiated for him. Studies suggest that SSRI is beneficial in paraphilic disorders via the primarily inhibiting effects on sexual functioning, leading to a delay in erection and ejaculation.

**Table 2:** Diagnosis and key diagnostic points

Diagnosis	Key diagnostic points
Exhibitionism	A recurrent urge to show one’s genitals to a stranger or an unknowing person whereby, in anticipation of the exposure, sexual excitement occurs [12]
Voyeurism	Scopophilia, or “peeping tom,”—is the recurrent preoccupation with fantasies and behaviors involving the observation of unsuspecting persons while naked or engaged in grooming or sexual activity [12]
Frotteurism	Characterized by a man’s rubbing his penis or hands against the body parts of a fully clothed woman to experience orgasm [12]
Compulsive sexual behaviors disorder (CSBD)	An impulse control disorder is characterized by a persistent pattern of failure to control intense, repetitive sexual urges and behaviors where an individual [13]: (1) devotes excessive time to sexual activities to the point of neglecting health, personal care, interests, and responsibilities (2) experiences diminished control manifest by multiple unsuccessful efforts to reduce sexual behaviors (3) continues sexual activity despite adverse consequences (4) continues engagement in sexual behaviors even when little or no satisfaction is derived (5) experiences significant distress or impairment across life domains or important areas of functioning

On top of the three DSM-5 diagnoses, there was an additional diagnosis of compulsive sexual behavior disorder, which is not in DSM-5 but is present under impulse control disorder in ICD-11. He presented with repetitive sexual behavior for several years, neglect of activities and responsibilities, and continuous behavior despite adverse consequences, resulting in impairment in social and occupational functioning.

It is essential to highlight this as the appropriate management must tackle not only paraphilia but also compulsive sexual behavior. The challenge in providing any interventions for this patient remains in that he continues to deny that any of his actions were causing him any trouble, despite potential legal implications. He also did not adhere to the medication prescribed despite psychoeducation on the possibility of it helping him return to his premorbid state.

## Discussion

Management of paraphilias is not extensively discussed in the scientific literature. This is likely because it is commonly secondary to some psychotic illness and thus treated accordingly. The DSM-5 lists eight specific paraphilic disorders, with the prevalence of exhibitionism estimated at around 2–4% in men, while voyeuristic disorder is around 12% in men [1]. Further complicating paraphilia is the need to distinguish it from nonparaphilic sexual fantasies and a whole battery of organic etiologies. Routine blood tests, brain imaging, infective screening, and specific tests of sexual organs, for example, penile strain gauge, Abel assessment for paraphilic interest, and phallometric testing, should ideally be performed [2].

Paraphilia is often comorbid with mood disorders, especially dysthymia (55%) and major depressive disorder (39%), and substance use disorder (40.8%) [3]. However, childhood attention deficit hyperactivity disorder is the only DSM-IV axis I disorder significantly associated with paraphilias [4]. The limited evidence for paraphilia treatment suggests that SSRI pharmacotherapy is the mainstay of biological management [5]. SSRI has been seen to decrease sexual fantasizing and desire in paraphilic fantasies [6]. It is also seen as helpful in decreasing obsessive–compulsive symptoms and increasing impulse control, which can be useful in those with compulsive sexual behaviors [7, 8].

Meanwhile, cognitive-behavioral therapy and brief psychodynamic psychotherapy have also helped manage paraphilia [9]. Management is essential for paraphilic disorders due to the high potential for crime and incarceration, as the motivation to change may fluctuate according to internal and external factors [10]. Unfortunately, there was no mandatory order for treatment in this case, as there were no prior convictions for the patient. Therefore, he will require better engagement by using motivational techniques to assess his motivation to engage in treatment and empower him to engage in treatment [11].

## Conclusion

Paraphilic disorders and compulsive sexual behaviors are very distressing to sufferers and their families alike, and thorough biopsychological investigations and comprehensive treatment are required to address the stigma and ensure maintenance in treatment to avoid potential legal issues that can arise.

## Data Availability

Not applicable.
